# Cytokines and Chemokines as Biomarkers of Community-Acquired Bacterial Infection

**DOI:** 10.1155/2013/190145

**Published:** 2013-04-16

**Authors:** Michal Holub, David A. Lawrence, Nancy Andersen, Alžběta Davidová, Ondřej Beran, Vilma Marešová, Pavel Chalupa

**Affiliations:** ^1^Department of Infectious and Tropical Diseases, First Faculty of Medicine, Charles University in Prague and Na Bulovce Hospital, Prague, CZ 180 81, Czech Republic; ^2^Laboratory of Immunology, Wadsworth Center, NYS DOH, Albany, NY 12201-0509, USA; ^3^Division of Infectious Diseases, Institute of Postgraduate Medical Education and Na Bulovce Hospital, Prague, CZ 180 81, Czech Republic

## Abstract

Routinely used biomarkers of bacterial etiology of infection, such as C-reactive protein and procalcitonin, have limited usefulness for evaluation of infections since their expression is enhanced by a number of different conditions. Therefore, several inflammatory cytokines and chemokines were analyzed with sera from patients hospitalized for moderate bacterial and viral infectious diseases. In total, 57 subjects were enrolled: 21 patients with community-acquired bacterial infections, 26 patients with viral infections, and 10 healthy subjects (control cohorts). The laboratory analyses were performed using Luminex technology, and the following molecules were examined: IL-1Ra, IL-2, IL-4, IL-6, IL-8, TNF-**α**, INF-**γ**, MIP-1**β**, and MCP-1. Bacterial etiology of infection was associated with significantly (*P* < 0.001) elevated serum concentrations of IL-1Ra, IL-2, IL-6, and TNF-**α** in comparison to levels observed in the sera of patients with viral infections. In the patients with bacterial infections, IL-1Ra and IL-8 demonstrated positive correlation with C-reactive protein, whereas, IL-1Ra, TNF-**α**, and MCP-1 correlated with procalcitonin. Furthermore, elevated levels of IL-1Ra, IL-6, and TNF-**α** decreased within 3 days of antibiotic therapy to levels observed in control subjects. The results show IL-1Ra as a potential useful biomarker of community-acquired bacterial infection.

## 1. Introduction

Rapid differentiation between viral and bacterial etiology of infection is necessary for decision on empirical antibiotic treatment. Furthermore, the initial antibiotic treatment cannot be switched to a pathogen-directed therapy in many patients, because the etiologic diagnosis of community- or hospital-acquired bacterial infections could not be confirmed or eventually is confirmed with a significant delay. Therefore, certain biomarkers such as C-reactive protein (CRP) and procalcitonin (PCT) are routinely used in clinical settings to help with both initial decision about antibiotic treatment and followup of its effect.

It must be stressed that CRP and PCT plasma levels are not 100% sensitive or specific, and these limitations hinder their clinical use. The major limitation of CRP is its low specificity in differentiating bacterial infection from autoimmune diseases and some hematological malignancies [1, 2]; CRP levels also are elevated by stress and cardiovascular disorders, which can be associated with metabolic syndrome [3]. Similarly, PCT is not a reliable biomarker of bacterial infection in patients with systemic inflammatory syndrome elicited by noninfectious causes, such as cardiopulmonary surgery and heat injury [4, 5]. Furthermore, an ideal biomarker of bacterial infection should be helpful in determining the efficacy of the antibiotic treatment. It is well known that the decline of CRP plasma level is a good predictor of the effectiveness of antibiotics, and the same opinion seems to hold true for PCT [6, 7]. On the other hand, both biomarkers have relatively long biological half-life, which is a problem that could be eliminated with the evaluation of inflammatory molecules that have short biological half-lives.

Thus, the aim of our study was to test serum levels of selected cytokines and chemokines in patients with community-acquired bacterial and viral infections and to compare them with concentrations obtained from healthy subjects. The kinetics of these molecules were also followed over the course of antibiotic therapy and assessed together with the changes of CRP and PCT plasma levels.

## 2. Patients and Methods

The study was approved by local ethics committee (IRB00002721), and it was executed in accordance with the Convention on Human Rights and Biomedicine (Oviedo 1997). The participants were enrolled in the study, only if they expressed their agreement by signing the informed consent. The clinical part of study was carried out in the Department of Infectious, Tropical, and Parasitic Diseases of University Hospital Bulovka between April 2007 and December 2009. In total, the study group consisted of 57 subjects: 21 patients with community-acquired bacterial infection, 26 patients with viral infections, and 10 healthy control subjects. The exclusion criteria consisted of antibiotic therapy instituted prior to the enrollment in the study, immunosuppression such as corticosteroid treatment, biological therapy or HIV infection, and autoimmune or malignant disease.

The diagnosis of bacterial infection was established by infectious diseases specialists based on clinical findings of focal infection associated with elevated CRP serum levels. Furthermore, bacterial pneumonia was confirmed by X-ray showing lobar infiltrate; urosepsis was confirmed with the detection of pathogenic bacteria in the blood, together with signs of urinary tract infection and pyelonephritis, with increased number of white blood cells in the urine and/or ultrasonography finding; diagnosis of erysipelas was established by characteristic skin signs, and toxic shock syndrome was confirmed based on the accepted clinical criteria. The etiology of bacterial infection was based on blood and/or urine cultivation, detection of pneumococcal antigens in urine, or positive serology in case of chlamydial infection. The specimens from patients with bacterial infection were collected at admission, on days 3 and 7. Demographic and clinical characteristics of this group are demonstrated in Table 1.

The diagnosis of viral infection was established based on characteristic clinical and laboratory findings, and it was confirmed by positive serology or detection of viral RNA. Notably, the diagnosis of Tick-borne encephalitis (TBE) was confirmed by detection of TBE specific IgM antibodies in the blood, similar to the laboratory diagnosis for acute hepatitis A (HA) or parvovirus infection.The diagnosis of enteroviral meningitis was confirmed with detection of viral RNA in cerebrospinal fluid. In addition, the diagnosis of varicella was based on characteristic skin findings of clear vesicles on the head and trunk, and the diagnosis of aseptic meningitis was made by exclusion of the most common viral and bacterial etiologies. In all patients from this study group, the viral etiology of infection was supported by low CRP serum levels and clinical improvement without antibiotic therapy. The demographic and clinical characteristics of this group of patients are demonstrated in Table 2.

For comparison, 10 healthy control subjects were enrolled in the study. These participants received prophylactic vaccines in the outpatient clinic of the department (nine received rabies vaccines and one received tetanus vaccine). This cohort consists of nine males and one female with mean age of 44.1 years and age range from 28 to 71 years. 

Venous blood from the study subjects was collected into S-Monovette (Sarstedt AG & Co., Nümbrecht, Germany) with serum separation gel in order to separate blood serum. All samples were centrifuged immediately after the collection, aliquoted, and stored at −80°C until further analyses were conducted.

Serum concentrations of CRP were measured on an AU 2700 analyzer (Olympus, Melville, NY, USA) using immunoturbidimetry (CRP Latex, Olympus) with a normal range of 0–8 mg/L. PCT levels were obtained using the enzyme-linked fluorescent assay technique. This assay combines a one-step immunoassay sandwich method with a final automatic fluorescent detection by a VIDAS instrument (VIDAS BRAHMS PCT, bioMérieux, Lyon, France). The measurement range of this instrument was set to 0.05–200 ng/mL.

The analyses of cytokines and chemokines were performed using a Luminex^100^ instrument (Luminex Corp., Austin, TX, USA) and R&D Systems Kit A and bead sets from R&D Systems (Minneapolis, MN, USA) to quantify the serum concentration of eight analytes: interleukin 1*β* receptor antagonist (IL-1Ra), interleukin- (IL-) 2, IL-4, IL-6, IL-8, tumor-necrosis-factor- (TNF-) *α*, macrophage-inflammatory-protein- (MIP-) 1*β*, and macrophage-chemoattractant-protein (MCP-) 1. The minimal detection concentrations (pg/mL) are as follows: IL-1Ra, 4.05; IL-2, 0.89; IL-4, 1.75; IL-6, 0.36; IL-8, 0.39; TNF-*α*, 0.60; MCP-1, 0.16; and MIP-1*β*, 0.44.

The statistical analysis employed for comparison among the study groups was one-way ANOVA test using software SigmaStat (Jandel Scientific, San José, CA, USA). Data are presented as mean ± standard deviation (SD) or as median (range).

## 3. Results

### 3.1. Serum CRP and PCT Levels of Patients with Bacterial and Viral Infections and of Control Subjects

The serum CRP concentrations (mean ± SD) of patients with bacterial infections (221.9 ± 65.2 mg/L) were significantly higher (*P* < 0.05) than those of the patients with viral infections (14.0 ± 13.8 mg/L) or of the controls (4.6 ± 7.0 mg/L). Similarly, the serum PCT concentrations of patients with bacterial infections (12.3 ± 22.5 ng/mL) were significantly higher (*P* < 0.05) than those of the patients with viral infections (0.12 ± 0.14 ng/mL) or of the controls (0.05 ± 0.01 ng/mL). 

### 3.2. Serum Cytokines and Chemokines Levels of Patients with Bacterial and Viral Infections and of Control Subjects

The serum IL-1Ra levels were detectable in all but two study subjects: control subjects (10 of 10; 100%), patients with a viral infection (25 of 26; 96.2%), and patients with a bacterial infection (20 of 21; 95.2%). With regard to the diagnostic potential of IL-1Ra serum levels, there were two patients with viral infections that had levels significantly higher (>20,000 pg/mL) than the median concentration of the patients with bacterial infections (11,618 pg/mL). The serum IL-2 levels were detectable in a majority of control subjects (9 of 10; 90%) and patients with a viral infection (20 of 26; 76.9%), whereas all patients with the community-acquired bacterial infections had detectable levels (21 of 21; 100%). For IL-6, serum levels were not detectable in any patient with a viral infection, in any control subjects, and in a minority of patients with a bacterial infection (5 of 21; 23.8%). The serum IL-8 levels were detectable in a majority of control subjects (8 of 10; 80%) but only in less than two-thirds of patients with a viral infection (16 of 26; 61.5%), whereas all the patients with the bacterial infections had detectable levels (21 of 21; 100%). The serum levels of TNF-*α* were detectable in a minority of control subjects (2 of 10; 20%) and patients with a viral infection (6 of 26; 23%), whereas the majority of patients with a bacterial infection had detectable serum TNF-*α* levels (18 of 21; 85.7%). Regarding the chemokines, the serum levels of MCP-1 were detectable in all but one study subject: controls (10 of 10; 100%), patients with a viral infection (25 of 26; 96.2%), and patients with a bacterial infection (21 of 21; 100%). The serum levels of MIP-1*β* were detectable in a majority of study subjects: controls (9 of 10; 90%), patients with a viral infection (23 of 26; 88.5%), and patients with community-acquired bacterial infections (21 of 21; 100%).

### 3.3. Comparison of Serum Cytokine and Chemokine Levels of Patients with Bacterial and Viral Infections and of Control Subjects

The concentrations of IL-1Ra, IL-2, IL-6, and TNF-*α* were significantly higher with the sera from the patients with bacterial infections than the sera from the patients with viral infections or the controls. Serum levels of MIP-1*β* were significantly higher with bacterial infection compared to the levels of control sera. Serum levels of MCP-1 were higher with bacterial infections than with viral infections. IL-4 was not detected in the sera of any patient or control subject, and IL-8 serum levels did not differ among the study cohorts. Data are shown in Table 3. There was no correlation of cytokine or chemokine levels of the virally infected patients with their age, length of stay in hospital, and extent of fever. However, for the patients with bacterial infection, there was positive correlation of the serum IL-6 levels with age (*r* = 0.479; *P* = 0.069) and duration of fever before admission to the hospital (*r* = 0.5; *P* = 0.056). In addition, there was no correlation with the length of stay in hospital. 

### 3.4. Correlations of Serum Levels of CRP, PCT, Cytokines, and Chemokines in Patients with Bacterial Infections

The serum CRP levels of patients with community-acquired bacterial infections, which were detected on admission to the hospital, correlated positively with their IL-1Ra (*r* = 0.611; *P* = 0.004) and IL-8 (*r* = 0.570; *P* = 0.007) serum levels. The serum levels of PCT of the patients with bacterial infections correlated positively with IL-1Ra (*r* = 0.725; *P* = 0.001), TNF-*α* (*r* = 0.659; *P* = 0.004), and MCP-1 (*r* = 0.556; *P* = 0.011). The serum IL-1Ra levels of the patients with bacterial infections correlated positively with IL-6 (*r* = 0.635; *P* = 0.014), IL-8 (*r* = 0.671; *P* = 0.001), TNF-*α* (*r* = 0.701; *P* = 0.001), MCP-1 (*r* = 0.671; *P* = 0.001), and MIP-1*β* (*r* = 0.614; *P* = 0.04). The serum TNF-*α* levels of the patients with bacterial infections correlated positively with IL-8 (*r* = 0.577; *P* = 0.012), MCP-1 (*r* = 0.556; *P* = 0.016), and MIP-1*β* (*r* = 0.738; *P* < 0.001) serum levels. The serum MCP-1 and MIP-1*β* levels of the patients with community-acquired bacterial infection correlated with each other (*r* = 0.435; *P* = 0.048).

### 3.5. Expression Kinetics of Cytokines and Chemokines during Antibiotic Therapy

The concentrations of IL-1Ra, IL-6, IL-8, TNF-*α*, and MIP-1*β* in the sera from the patients with bacterial infections were significantly elevated at admission, and the majority of the patients' levels subsequently decreased to the levels of the controls by day 3. Conversely, the IL-2 serum levels were persistently elevated over the study period in comparison to controls. The serum levels of IFN-*γ* and MCP-1 did not significantly differ from the concentrations of the control subjects over the study period. Data are demonstrated in Table 4.

## 4. Discussion

In this study, selected inflammatory cytokines and chemokines were evaluated as potential biomarkers of community-acquired bacterial infections. Additionally, their expression kinetics during antibiotic therapy and correlations with CRP and PCT were tested.

The highest serum concentrations from analyzed cytokines and chemokines were those of IL-1Ra. Elevated serum levels of this anti-inflammatory molecule were already demonstrated in septic shock patients in whom the high concentrations correlated positively with a fatal outcome [8]. Olszyna et al. [9] reported the median value of IL-1Ra in the sera of patients with urosepsis to be 7,400 pg/mL, which was significantly higher than the median level of healthy controls (410 pg/mL). Similar findings were observed in the present study with the highest IL-1Ra levels detected in three patients with severe bacterial infections: toxic shock syndrome, urosepsis, and community-acquired pneumonia (CAP). In contrast to the study by Olszyna et al., the high IL-1Ra concentrations in the present study were not associated with positive blood cultures, and the lowest concentration was found in a patient with pneumococcal bacteremia (data not shown). It is of interest to note that increased IL-1Ra serum levels were also demonstrated in patients with fulminant hepatic failure (FHF) and acute viral hepatitis [10]; the highest IL-1Ra serum levels (i.e., 7,208 and 6,930 pg/mL) were observed in two patients that survived FHF. Increased serum concentrations of IL-1Ra were reported in adult and pediatric patients with infection due to dengue virus and also in some patients with parvovirus [11]. Interestingly, from the cohort of patients with viral infections enrolled in the present study, the highest IL-1Ra serum levels were detected in the patient with acute HA and the patient with parvovirus (data not shown), but currently there is no explanation for the very high levels in these two patients. 

Regarding IL-6 and TNF-*α* serum levels, the data of the present study are in agreement with previous studies, which demonstrated important limitations of these two inflammatory cytokines as biomarkers of bacterial infection. A notable problem with implementation of TNF-*α* as a routine diagnostic biomarker is its undetectable serum level in a significant percentage of patients with a proven Gram-positive bacterial infection of moderate disease severity [12]. Similar to TNF-*α*, low or undetectable IL-6 serum levels are reported for some patients with severe bacterial infections, which limits its usefulness for ruling out sepsis and bacteremia [13]. IL-6 levels are often below detectable limits, but IL-6 is considered a stress-related proinflammatory cytokine that usually correlates with CRP and is often used as a biomarker of acute and chronic inflammation [14–16]. It is worth noting that the high IL-6 serum levels are capable of predicting the severity of bacterial infection, because they are associated with the development of systemic inflammatory response and progression to shock [17, 18]. Despite all of these limitations, elevated IL-6 serum levels may help to differentiate bacterial and viral infections, although serum levels of PCT, cortisol, and heparin-binding protein have been demonstrated to be of greater diagnostic value for community-acquired infections [19].

The majority of the elevated cytokines and chemokines (i.e., IL-1Ra, IL-6, IL-8, TNF-*α*, and MIP-1*β*) in the patients with community-acquired bacterial infections decreased to the median concentrations detected in controls within 3 days of the study period. The most pronounced decrease was demonstrated with serum IL-1Ra levels, a decline that has already been reported for patients with CAP during antibiotic therapy, and similar kinetics were observed with sera from patients having an invasive meningococcal disease, who were treated with third generation cephalosporins [20, 21]. For the followup of antibiotic treatment, serum IL-6 levels seem to be the most often studied cytokine. Rapid decline of IL-6 was demonstrated in the sera of females with acute pyelonephritis due to *Escherichia coli, *who were treated with broad spectrum antibiotics [22]. Furthermore, lower serum IL-6 levels were observed in patients with CAP, who were pretreated with antibiotics before enrollment in the study, in comparison to patients who received antibiotics after the collection of serum samples [23]. There is a lack of data concerning the kinetics of MIP-1*β* and IL-8 in sera during community-acquired infections; however, in the previously mentioned study of patients with *E. coli *pyelonephritis, TNF-*α* levels were already elevated 6 hrs after the administration of antibiotics [22].

Profiles of cytokines and chemokines demonstrated in the present study indicate that proinflammatory and anti-inflammatory responses are activated simultaneously. This finding supports the hypothesis that systemic inflammatory response may be associated with suppression of immune functions [24]. Systemic proinflammatory responses can be characterized in the enrolled patients by elevated serum levels of TNF-*α*, IL-2, IL-6, MCP-1, and MIP-1*β* [25, 26]. As mentioned above, the kinetics of these molecules showed rapid decline with the exception of IL-2, which is necessary for proliferation and differentiation of effector T lymphocytes. Thus, the persistent elevation of IL-2 serum levels may indicate activation of adaptive immunity in spite of lymphocytopenia, which frequently accompanies bacterial infections [27]. The major anti-inflammatory molecule detected was IL-1Ra, which can effectively neutralize proinflammatory effects of IL-1*β* [25]. However, in an experimental model, it was shown that IL-1Ra attenuates the intensity of inflammation induced by proinflammatory cytokines, but it was not able to modulate inflammation induced by the whole bacteria or its lipooligosaccharide [28]. 

The present study has several limitations. First, both cohorts of patients were not homogenous enough; it might have been more appropriate to compare viral and bacterial infections affecting the same anatomic site, such as respiratory tract or central nervous system. On the other hand, the primary aim of the present study was evaluation of biomarkers of community-acquired infection, which was reflected in the study design. Second, the cohort of controls included patients that received prophylactic vaccination, which could lead to increased serum levels of inflammatory cytokines and chemokines; significant increases of IL-6, TNF-*α*, and CRP were shown after flu vaccine [29]. Third, concentrations of IL-1Ra in the sera of enrolled patients were higher than in similar studies; however, the levels were comparable with the data previously published by our group [21]. Finally, the etiological diagnosis of bacterial infection was confirmed only in 43% of patients enrolled in the bacterial infection cohort; however, a similar percentage of etiological diagnosis was observed even in a study with septic patients combining regular cultivation and molecular biology techniques [30]. 

## 5. Conclusion

The results indicate several alternative biomarkers of bacterial infection, from which IL-1Ra seems to be the most promising. However, future prospective studies with large cohorts of patients are necessary in order to implement a new biomarker in routine laboratory testing.

## Figures and Tables

**Table 1:** Demographic and clinical characteristics of patients with bacterial infection.

Parameters	Bacterial infection
	*n* = 21
Demographic characteristics	
Years of Age (mean; range)	48; 18–85
Sex (female/male)	11/10
Clinical characteristics	
Fever (days; mean ± SD)	3 ± 3
Hospitalization (days; mean ± SD)	12 ± 6
Diagnosis	
Bacterial pneumonia	9
Pyelonephritis	6
Erysipelas	3
Urosepsis	2
Toxic shock syndrome	1
Etiologic agents	
* Escherichia coli *	5
* Streptococcus pneumoniae *	2
* Chlamydophila pneumoniae *	1
* Streptococcus agalactiae *	1

**Table 2:** Demographic and clinical characteristics of patients with viral infection.

Parameters	Viral infection
	*n* = 26
Demographic characteristics	
Years of Age (mean; range)	43.2; 19–69
Sex (female/male)	9/17
Clinical characteristics	
Fever (days; mean ± SD)	5.4 ± 4
Days in hospital (mean ± SD)	11.2 ± 3.6
Diagnosis	
Tick-borne encephalitis	13
Enteroviral meningitis	4
Aseptic meningitis	4
Varicella	2
Hepatitis A	1
Parvovirus	1

**Table 3:** Serum cytokine and chemokine levels.

Analyte (pg/mL)	Bacterial infection (*n* = 21)	Viral infection (*n* = 26)	Control subjects (*n* = 10)	*P* value
IL-1Ra	15,868 ± 12,697*	4,090 ± 5,543	2,376 ± 2,563	<0.001
IL-2	22.2 ± 9.3*	11.2 ± 8.5	11,3 ± 7.2	0.002
IL-4	n.d.	n.d.	n.d.	n.t.
IL-6	237.4 ± 408.6*	n.d.	n.d.	<0.001
IL-8	49.3 ± 75.1	12.5 ± 15.2	12.1 ± 15.7	n.s.
TNF-*α*	20.7 ± 17.1*	3.0 ± 5.2	3.6 ± 8.1	<0.001
MCP-1	853.3 ± 1,175^#^	156.6 ± 104.4	223.3 ± 88.7	0.007
MIP-1*β*	222.1 ± 374.7^+^	56.0 ± 37.4	53.4 ± 26.4	0.005

Data are presented as mean ± standard deviation; n.d.: not detected; n.s.: not significant; n.t.: not tested; *P < 0.05, bacterial versus viral infection and control; ^#^ *P* < 0.05, bacterial infection versus viral infection only; ^+^ *P* < 0.05, bacterial versus control only.

**Table 4:** Kinetics of inflammatory molecules in patients with bacterial infection during antibiotic therapy.

Analyte (pg/mL)	Day 1	Day 3	Day 7	Control	*P* value
IL-1Ra	11,618*	3,247	2,604	1,544	<0.001
	(42,790)	(11,492)	(8,607)	(8,768)	
IL-2	22.5*	21.4*	19.5*	11.9	<0.013
	(36.5)	(57.4)	(71.2)	(26.1)	
IL-6	39.1*	1.9	0.4	0.4	<0.001
	(1,597)	(150)	(95.8)	(0.0)	
IL-8	26.6*	21.2	14.6	6.9	<0.039
	(350)	(648)	(58)	(54)	
TNF-*α*	10.8*	5.5	4.5	0.6	<0.020
	(61.0)	(31.2)	(16.8)	(25.8)	
MCP-1	326	187	231	205	n.s.
	(7,524)	(210)	(287)	(279)	
MIP-1*β*	91.0*	76.8	60.4	54.6	<0.025
	(1,699)	(275)	(161)	(91.2)	

Data are presented as median (range); n.s.: not significant; *P < 0.05, time point versus control.
